# In Silico Prediction of Cross-Reactive Epitopes of Tropomyosin from Shrimp and Other Arthropods Involved in Allergy

**DOI:** 10.3390/molecules27092667

**Published:** 2022-04-21

**Authors:** Jirakrit Saetang, Varomyalin Tipmanee, Soottawat Benjakul

**Affiliations:** 1 International Center of Excellence in Seafood Science and Innovation, Faculty of Agro-Industry, Prince of Songkla University, Hat Yai, Songkhla 90110, Thailand; soottawat.b@psu.ac.th; 2 EZ-Mol-Design Laboratory, Faculty of Medicine, Prince of Songkla University, Songkhla 90110, Thailand; tvaromya@medicine.psu.ac.th; 3 Department of Biomedical Sciences and Biomedical Engineering, Faculty of Medicine, Prince of Songkla University, Songkhla 90110, Thailand

**Keywords:** shellfish allergy, tropomyosin, cross-reactivity, house dust mite, cockroach, epitope

## Abstract

Tropomyosin in shellfish is considered a major cross-reactive allergen in house dust mites and cockroaches; however, the specific epitopes have not been elucidated. Therefore, this study aimed to identify the consensus antigenic determinant among shrimp, house dust mites, and cockroaches using in silico methods. The protein sequences of tropomyosin, including Der f 10, Mac r 1, Pen a 1, Pen m 1, Per a 7, and Bla g 7, were retrieved from the UniProt database. The 3D structures were derived from the AlphaFold or modeled using the Robetta. The determination of linear epitopes was performed by AlgPRED and BepiPRED for B cell epitope, and NetMHCIIpan and NetMHCII for T cell epitope, while Ellipro was used to evaluate conformational epitopes. Fourteen peptides were discovered as the consensus linear B cell epitopes, while seventeen peptides were identified as linear T cell epitopes specific to high-frequency HLA-DR and HLA-DQ alleles. The conformational determination of B cell epitopes provided nine peptides, in which residues 209, 212, 255–256, and 258–259 were found in both linear B cell and linear T cell epitope analysis. This data could be utilized for further in vitro study and may contribute to immunotherapy for allergic diseases associated with tropomyosin.

## 1. Introduction

Seafood is considered one of the “big eight” food groups responsible for 90% of all food allergies and anaphylaxis worldwide [1]. Nowadays, about 2.5% of the world’s population has been affected by the adverse reaction from a shellfish allergy, particularly in the regions with high seafood consumption [2]. Individuals with a shellfish allergy are susceptible to the consumption of crustaceans, such as crab or shrimp, or mollusks, such as clams or scallops, or both [3,4]. The allergic reaction mainly mediated by IgE includes mouth and throat itching, lip swelling, urticaria, periorbital angioedema, and skin redness [5]. These symptoms may vary from instant response to late-phase reactions up to 8 h after allergen consumption [5].

Although many natural proteins found in shellfish have been identified as allergens, the muscle protein, namely tropomyosin, seems to play the dominant role since IgE antibodies from 60–80% of shellfish allergic patients recognized this protein [6,7]. Moreover, this protein has been identified as the most important allergen of shrimp since IgE antibody from 72–98% of shrimp-allergic patients binds to the purified tropomyosin [8,9,10]. Tropomyosin is a heat-stable protein with an alpha-helical coiled-coil dimeric structure [11]. The protein consists of 276–284 amino acids with a 34–38 kDa molecular weight, depending on the species [2]. The primary structure of tropomyosin is highly conserved, which causes this protein to be a significant allergen over 14 crustacea and five mollusk species [12]. Since this protein plays a vital role in controlling the contraction of muscle fibers, it can be identified in over 150 species, including insects and mites [13].

Interestingly, allergy to shellfish is associated with other arthropods, such as house dust mites and cockroaches [14]. A study showed that 72.4% of atopic Singaporean children who are allergic to shellfish are also sensitized to house dust mites [15]. Cross-reactivity between shrimp and house dust mite was also demonstrated in 19.4% of house dust mite-associated allergic rhinitis patients. Nevertheless, almost half (41.2%) did not previously consume shrimp. Asthmatic children from the U.S. is another case study that showed a strong positive correlation between shrimp and house dust mite IgE levels [16].

Some researchers highlighted that tropomyosin plays a critical role in cross-reactivity between house dust mites and shellfish. Boquete et al. [17] discovered that more than 70% of house dust mite allergic patients had specific IgE antibodies to shrimp, and 55% of them had IgE specific to shrimp allergen tropomyosin. Another study also demonstrated that mite tropomyosin-specific IgE strongly reacted to shrimp tropomyosin, although it was found at very low levels in house dust mites [18]. This cross-reactivity may be explained by the fact that tropomyosin of house dust mites and cockroaches have 80–82% sequence identity to shrimp tropomyosin (Pen a 1) [19]. It is hypothesized that sensitization to tropomyosin can occur through the consumption of shellfish or through the inhalation of mites, and sensitization to either of these can trigger an allergy to both animals and vice versa [20].

Although the protein sequence and sensitization capacity of tropomyosin has been well studied, little information about the immunological mechanism of this protein, which may be related to hypersensitivity mediated by IgE response and cross-reactivity between shrimp, house dust mites, and cockroaches, exists. Therefore, this study aimed to elucidate cross-reactive epitope among these arthropods related to allergy, especially from high seafood consumption countries, using an in silico approach.

## 2. Results

### 2.1. Sequence Retrieval and Analysis

**Figure 1:**
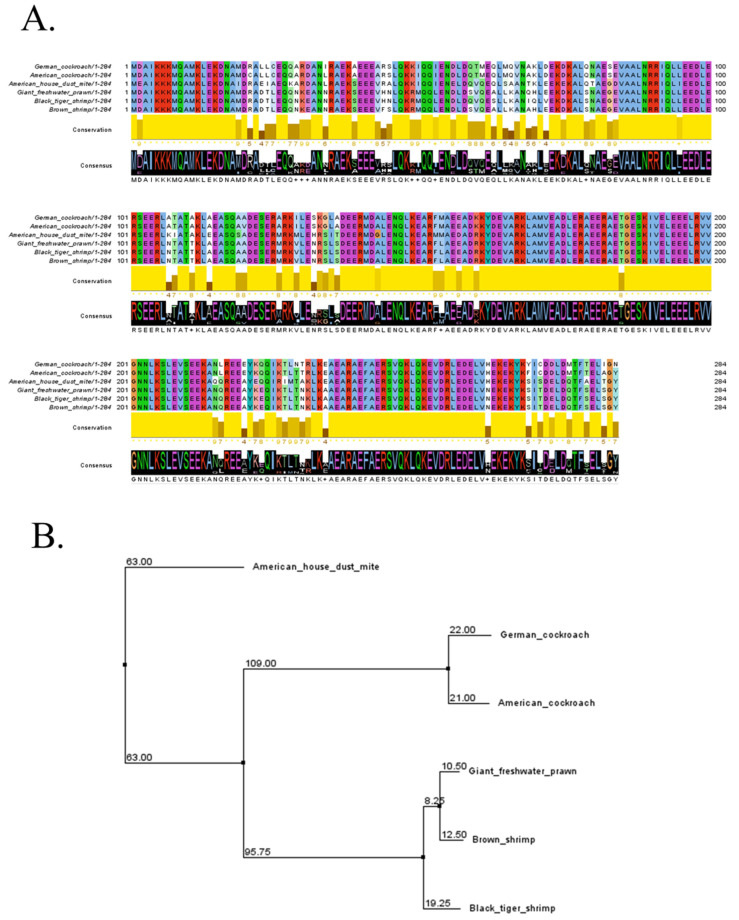
Multiple sequence alignment of tropomyosin from different species selected in this study. (**A**) Protein sequences of tropomyosin from selected species were aligned using Clustal Omega. The conservation of amino acids was indicated as the conservation bar. The consensus sequences were also demonstrated. (**B**) Phylogenetic tree derived from BLOSUM62 matrix based on tropomyosin amino acid sequences.

Amino acid sequences of tropomyosin were selected from three shrimp species, two cockroaches, and American house dust mites that have been reported for their cross-reactivity [14,17,18]. This included giant freshwater prawn, black tiger shrimp, brown shrimp, German cockroach, American cockroach, and American house dust mite (Table 1). All sequences were utilized for multiple alignments using the Clustal Omega server. The main four regions of tropomyosin were found to be highly conserved among six animals (Figure 1A). The shortest sequence was located close to the N-terminus, including residues 2–18 and 85–106. The longest identical sequence included residues 161–214, which constituted around 53 amino acids of the tropomyosin sequence. The last one was found to be residues 235–261 close to the C-terminus. All these conserved amino acids account for more than one-third of the sequence of tropomyosin. Phylogenetic analysis demonstrated three nodes of relationship among tropomyosin from six species (Figure 1B). Firstly, tropomyosin of the American house dust mite showed a highly distinctive relationship with other animals. Meanwhile, American cockroaches and German cockroaches are members of the second node. Giant freshwater prawn, black tiger shrimp, and brown shrimp were included in the third node with a close relationship.

### 2.2. Identification of Possible Cross-Reactive Linear B Cell Epitopes

**Figure 2:**
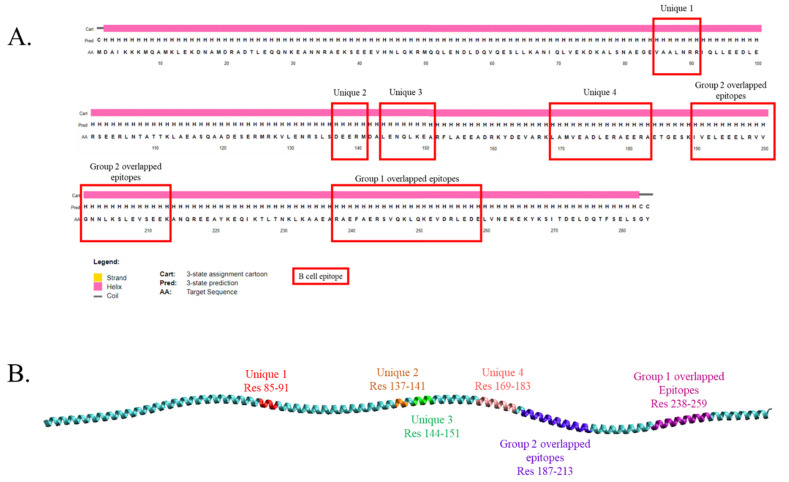
Consensus linear B cell epitope identified by AlgPRED and BepiPRED web tools. (**A**) Sequence with the predicted secondary structure of black tiger shrimp tropomyosin. The predicted consensus amino acids responsible for cross-reactive allergy were indicated in the red square. (**B**) The location of each type of identified linear B cell epitopes illustrated in 3D structure of black tiger shrimp tropomyosin derived from AlphaFold database.

Most allergens contain epitopes that are specific to IgE antibodies, as this is crucial to determine the allergic reactions. In order to identify the possibly cross-reactive IgE epitopes of tropomyosin from animals studied, the AlgPRED tool was used to predict the possibility of the protein being allergenic, and IgE epitope motifs were extracted from the sequence based on known allergens. In addition, the candidate IgE epitope was analyzed using the BepiPRED server for cross-validation. When the AlgPRED tool was applied for all tropomyosin sequences of all species in the present study, more than fifty peptides were identified to be IgE epitopes. However, only fifteen peptides were discovered as the consensus sequences for all species (Table 2). All peptides were found to be both IgE epitopes and B cell epitopes except L169AMVEADLERAEERA183, which was not a B cell epitope in giant freshwater prawns analyzed by BepiPRED (Table 2). Some peptides were overlapped with the different lengths and regions, such as peptides B1 (residues 238–252), B2 (241–255), B3 (243–259), B4 (244–258), and B5 (251–259) as group one of the overlapped epitope, and peptides B6 (residues 187–197), B7 (190–204), B8 (193–207), B9 (196–210), B10 (199–206), and B11 (199–213) as group two (Figure 2A,B). Only four peptides were categorized as unique sequences and did not overlap with any consensus residues, including V85AALNRR91, D137EERM141, L144ENQLKEA151, and L169AMVEADLERAEERA183.

### 2.3. Identification of Cross-Reactive T Cell Epitope Candidates

Helper T cell plays a major role in contributing to the physiological changes during food allergy. This scenario needs the presence of a food epitope mediated by antigen-presenting cells (APCs) via MHC class II molecules [21] for allergen response. Therefore, NetMHCII 2.3 and NetMHCIIpan 4.0 servers were adopted to predict the possible consensus T cell epitopes of tropomyosin between animals selected in this study. The results showed that nineteen peptides specific to six HLA-DQ alleles were found to be MHC class II binders by using the NetMHCII tool (Table 3). HLA-DQA10301-DQB10302 contained the highest number of members with seven types of core epitopes, while DQA10102-DQB10602 had only one consensus peptide of tropomyosin (A166RKLAMVEA174). DQA10401-DQB10402 and DQA10501-DQB10201 alleles showed the second and third lower number of tropomyosin binding epitopes, including residues 169–177, 186–194, 188–196, 165–173, 187–195, and 204–212 for the former allele and residues 169–177, 188–196, 169–177, 189–197, and 253–261 for the latter allele. However, no consensus epitope was identified for DQA10101-DQB10501 and DQA10501-DQB10301 alleles. In addition, ten core epitope sequences were identified to be specific to HLA-DR alleles. DRB4*0101 allele could be bound to five types of peptides, while the number of tropomyosin epitopes of DRB1*0301 and DRB5*0101 alleles was three and two peptides, respectively. In conclusion, the T cell epitope sequence covered around one-fourth of tropomyosin amino acids, including the regions of residues 8–16, 144–152, 165–181, 186–212, and 253–261. Moreover, seventeen unique peptides were found to be the possible cross-reactive epitopes of MHC-II alleles and could be categorized as the seven strong binders and the ten weak binders (Table 3).

### 2.4. Structural Modeling, Refinement and Validation

**Figure 3:**
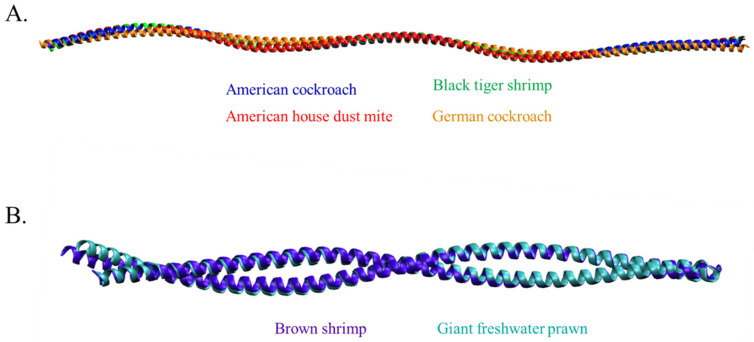
Predicted tropomyosin tertiary structures. (**A**) The comparison of tropomyosin structure derived from American cockroach, black tiger shrimp, American house dust mite, and German cockroach (α-helical coiled-coil along the entire molecule). (**B**) The unnatural structure of tropomyosin predicted by Robetta server (self-assembly alpha parallel molecule).

No tropomyosin in this study was resolved experimentally, and there are three types of tropomyosin (Der f 1, Pen m 1, and Per a 7) from three species that have been found in the AlphaFold database, the deep machine learning algorithm that demonstrated the highest accuracy of protein structure prediction among other servers [22]. The structures of Bla g 7, Mac r 1, and Pen a 1 were modeled using the Robetta protein structure prediction server with the RoseTTAFold algorithm, a deep learning-based method that showed the top-ranked in CAMEO and CASP14 [22]. Predicted model 1 of all allergens, which displayed the lowest mean angstrom error estimate, was selected for structural refinement together with structures retrieved from AlphaFold. The GalaxyRefiner2 server was used with an iterative optimization approach for both local and global protein refinement, in which the accuracy of the structure could be improved [23]. As demonstrated in Figure 3A, the conformation of tropomyosin of the American cockroach, American house dust mite, German cockroach, and black tiger shrimp showed a well-known elucidated coiled-coil structure. However, the model of brown shrimp and giant freshwater prawn derived from Robetta displayed an uncommon appearance since the end region sequences formed self head-to-tail interaction and winding of two α-helices along the molecule (Figure 3B).

The refined models were subjected to quality validation using a Ramachandran plot analysis derived from PROCHECK [24]. The result showed that the favorable region of all predicted tropomyosin structures was 100%, while the residues in both the allowed region and residues in the outlier region displayed 0% (Table 4). This indicated the reliability of the refined models since more than 85% of residues were in the favored region. The ERRAT online server was also used to verify the statistics of non-bonded interactions between different atom types. The results showed that all structures of tropomyosin provided an overall quality factor of 100 (Table 4), indicating that all tertiary structures of tropomyosin had high resolution. The Z-score of the 3D models derived from ProSA analysis had the values within the range commonly found for proteins of 284 amino acid residues deposited in the PDB. In addition, the QMEAN server with the QMEANDisCo method was applied to all computationally refined structures for qualitative model energy analysis [25]. The results showed that the QMEAN score of the refined structure was in the range of 0.66–0.82, and the best score was attained for American and German cockroaches, suggesting the good quality of overall folding and local structure. For the QMEAN score below 0.7, the downstream analysis was truncated.

### 2.5. Discontinuous B Cell Epitope Prediction

**Figure 4:**
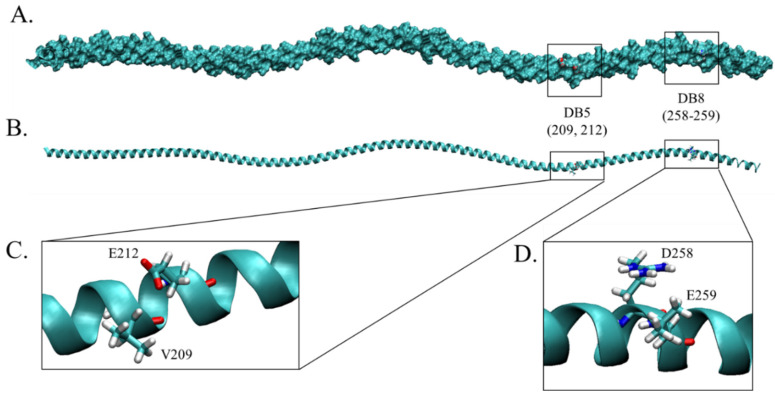
Cross-reactive epitopes identified by the linear B cell, linear T cell, and discontinuous B cell epitope analysis. (**A**) The structure of black tiger shrimp illustrated as surf conformation. The possible cross-reactive epitopes with high affinity to T cell MHC molecules were indicated in the square. (**B**) The new cartoon structure of tropomyosin with the predicted cross-reactive epitopes. (**C**) The identified epitope residues glutamic acid (E) and valine (V) at positions 212 and 209, respectively, in DB5 epitope. (**D**) The continuous amino acids aspartic acid (D258) and glutamic acid (E259) were illustrated as part of epitope DB8.

After validation and quality evaluation of the tropomyosin structures, the Ellipro web tool was further employed to predict conformational epitopes of the refined tropomyosin structures. It was noted that brown shrimp and giant freshwater prawn were excluded from this analysis according to the QMEANDisCo score. In addition, the results were categorized as cross-reactive discontinuous B cell epitopes when the consensus of residue numbers was discovered without considering residues sequences. As shown in Table 5, nine consensus epitopes were identified using Ellipro. The lengths of predicted cross-reactive conformational epitopes ranged from 3 to 16 mers. Some of them demonstrated the consensus amino acids, including DB5 (V209, E212K213) and DB7 (A237R238, E240, R244S245, K248L249, E252, R255L256) epitopes, while DB8 and DB9 covered the region that varied in amino acid compositions. The region DB1 displayed inconsistency in amino acid sequences but conserved in polarity according to the Clustal X color scheme (Figure 1A) [26]. Moreover, when compared to the results from linear B cell and linear T cell epitope analysis, the residues 209, 212, 255–256, and 258–259 were identified as the antigenic determinant region of tropomyosin in all kinds of the epitope. Among these, only amino acids at positions 208, 212, and 258–259 were high-affinity T cell epitopes (strong binders) (Figure 4).

## 3. Discussion

Shellfish sensitization is a common reaction found in 1–2% of the U.S. population, while 5% of South-East Asian individuals are affected [27,28,29]. Tropomyosin, a heat-stable muscle protein, is recognized as a major allergen responsible for shellfish and seafood allergies [4,13]. This protein is highly homologous, commonly found in most edible crustaceans and other arthropods, including cockroaches and dust mites [2]. Therefore, many cross-sectional studies revealed a significant association between shellfish, house dust mite, and cockroach sensitization [16,30]. Moreover, the results confirmed that tropomyosin contained the conserved amino acids that demonstrated a high similarity with more than 80% identity among crustaceans and invertebrate species selected in this study. Other works also reported that IgE from house dust mite allergic patients had the capability to react with purified shrimp tropomyosin [17,18], reconfirming the role of this protein in allergic cross-reactivity. However, although tropomyosin was suggested as the major cause of shrimp, house dust mite, and cockroach allergic cross-reactivity, the possible epitopes responsible for this condition have not been revealed. Therefore, this is the pioneer study that tried to explore both cross-reactive B cell and T cell epitopes using in silico methods, which may be beneficial for vaccine development in allergen-specific immunotherapy.

Cross-reactive epitopes of tropomyosin from shellfish (shrimps), house dust mites, and cockroaches were identified. These arthropod species have been reported as the main cross-allergic reaction in shellfish sensitized patients [2,12,16,18,19,31]. Therefore, three types of shellfish and three arthropods were initially selected. For shellfish, black tiger shrimp and giant freshwater prawn were chosen because they are the main cultured species with economic importance, accounting for more than 9% of the crustacean market [32]. Brown shrimp was included based on its natural habitat in the environment with its socio-economic importance for the fishery economy in the North Sea [33]. For arthropods, the American house dust mite was selected since this kind of mite is a widely distributed species that provides the major source of house dust mite tropomyosin allergen [34]; American and German cockroaches were used since they are ubiquitous, difficult to control, predominant, and often correlated with allergy and asthma [35].

Currently, the possible linear B cell epitopes responsible for the allergic cross-reactivity between Der f 10, Mac r 1, Pen a 1, Pen m 1, Per a 7, and Bla g 7 were identified via in silico prediction based on the protein sequences. These peptides were not only possibly antigenic for B cells but also specific to IgE antibodies, suggesting that all identified sequences were associated with allergies. Interestingly, most predicted tropomyosin epitopes (11/14) had been previously proven for their antigenicity in other crustacean species by using inhibitory dot-blot analysis and patient serum [36,37,38]. It is necessary to note that peptides B5, B7, B12, and B13 were reported as the consensus results of candidate epitopes in four reports [36,37,38,39]. Moreover, half of these peptides were fully or partially LEXXL motif-containing epitopes (B5 and B7), where L is leucine, E is glutamate, and X is D, E, N, or Q. This kind of tandem was often found in other allergens, such as latex [40] or helminths [41]. Furthermore, the B12 sequence (V85AALNRR91) was found to strongly react to IgE antibodies of more than 50% of shellfish allergic patients [37,38,39]. With this fact, peptide B12 may mainly contribute to multisensitization and symptoms in individuals who are allergic to shellfish, house dust mites, and cockroaches.

The presentation of allergens was considered the first step of allergic reaction. Naive CD4 T cells recognize epitope via HLA class II-mediated antigen binding and transform into CD4 memory T cell that subsequently stimulates the differentiation of B cell to plasma cell, leading to the production of allergen-specific IgE antibodies. Therefore, the identification of T cell antigenic determinants is also essential for the treatment of allergy. For shellfish T cell epitope, Ravkov et al. [42] experimentally explored CD4 specific T cell epitopes of tropomyosin, in which sequences were in line with the present results. Seventeen peptides from shrimp-tropomyosin that positively reacted to CD4 T cell tested by both the MHC binding and CD4 T cell proliferation assay was identified. Interestingly, more than half of the strong binders (peptides T05, T15, T16, and T17) found in the present study also appeared in their results. Moreover, the present results demonstrated the cross-reactive T cell epitopes between Der f 10, Mac r 1, Pen a 1, Pen m 1, Per a 7, and Bla g 7, corresponding to certain HLA-DR and HLA-DQ alleles that were found more than 10% in the human population [43]. Among seven high-affinity epitopes, six (T02, T03, T05, T07, T15, and T17) were observed to overlap with predicted B cell epitopes, indicating that these sequences may be important for all types of both B cell and T cell response. Furthermore, T02 and T03 epitopes showed the specificity to the highly distributed HLA DQ allele, DQA1*301–DQB1*302, which may make this sequence the cause of sensitization in most people. Together with the results, these sequences also partially overlapped with the highly prevalent consensus B cell epitope, B7; this emphasized the role of B7 peptides in the epidemiological spreading of tropomyosin allergy.

As described above, no crystal structure has been experimentally resolved for all tropomyosin in this study, although this allergen has been realized for its allergenicity for a long time. Fortunately, three tropomyosin from three species, including Der f 1 (American house dust mite), Pen m 1 (black tiger shrimp), and Per a 7 (American cockroach), were predicted for their structures in the AlphaFold database, the server that showed the rank 1 of accuracy testing for protein structure modeling [22]. For the remaining allergens, the Robetta server with the RoseTTAFold algorithm, that has been reported to provide highly accurate predicted models, was used for the remaining allergens (German cockroach, Bla g 7; Giant freshwater prawn, Mac r 1; Brown shrimp, Pen a 1). This server has successfully generated the models for multipurpose scientific research; for example, Tan et al. [44] used Robetta to de novo model Cache domains of nine genes of bacteria Geobacter sulfurreducens for studying the regulation of cGAMP. Another study also predicted the structure of the M protein of the porcine epidemic diarrhea virus and used this model to identify both B cell and T cell epitopes for the treatment development purpose [45]. Although the tropomyosin structures from different species were derived from the two methods of modeling, all structures were subjected to the same protocol of refinement (GalaxyRefine2) before performing the quality assessment using several methods, including ERRAT, PROCHECK, ProSA, and QMEAN, to ensure that all models passed the same standard protocols of accuracy checking in both local and global coordinates.

An attempt to use the outperform online tools according to the standard testing benchmark was made; however, the tropomyosin structures of brown shrimp and giant freshwater prawn revealed the uncommon conformation with the low quality according to the QMEANDisCo score and Ramachandran plot. Although the bending form of tropomyosin had been suggested in crystallized analysis [46,47], the self-assembly structure predicted by Robetta is not the natural form of this protein since most of the literature mentioned the structure of tropomyosin as a parallel α-helical coiled-coil along the entire molecule [48]. From this data, tropomyosin of these two species was excluded from further analysis and used only the high score structures for discontinuous epitope identification. It was illustrated that certain residues found in this analysis were categorized as allergenicity in accordance with the results from linear B cell and T cell epitope analysis. The residues of 255–256 and 258–259 of epitopes DB7 and DB8 were not only discovered as both predicted linear B cell and linear T cell epitopes but also had experimental evidence, suggesting that these amino acids were responsible for allergic reactions [36,37,39,42].

This work mostly explored the possible cross-reactive tropomyosin epitopes between shellfish, house dust mite, and cockroaches based on experimental evidence from the literature on allergic patients. This study and other findings demonstrated a strong allergic reaction between shellfish and insects, which may raise an issue of concern about the promotion of eating edible insects as an alternative source of nutrients [49,50,51]. It should be noted that there were some limitations since all results were from the in silico evaluations, which may not be the exact information. Moreover, the epitopes proposed in the present study need further confirmation through experimental studies so that the data will be useful for the development of rational strategies in the diagnosis and therapy of patients.

## 4. Materials and Methods

### 4.1. Sequence Retrieval and Analysis

The data on allergen names and sources were obtained from the World Health Organization (WHO)/International Union of Immunological Societies (IUIS) Allergen Nomenclature Sub-committee database (http://allergen.org/; accession date: 12 January 2022). The amino acid sequences of tropomyosin from 6 animals (Der f 10, Mac r 1, Pen a 1, Pen m 1, Per a 7, and Bla g 7) were acquired from The Universal Protein Resource (UniProt) according to the accession number (Table 1). The protein sequences of all allergens studied were aligned using Clustal Omega [26]. The phylogenetic tree was obtained using the BLOSUM62 matrix, based on the likelihood that two amino acids would match by random chance [52].

### 4.2. Prediction of Linear B Cell Epitopes and IgE Epitopes

Epitopes of the allergens were predicted based on the selected parameters, including the mapping of IgE epitopes and linear B-cell epitope mapping of motifs. Two immunoinformatics tools, including AlgPRED 2.0 (https://webs.iiitd.edu.in/raghava/algpred2; accession date: 10 February 2022) and BepiPRED-2.0 server (https://services.healthtech.dtu.dk/service.php?BepiPred-2.0; accession date: 10 February 2022) were used to predicate the cross-reactive epitopes. AlgPred employs a wide range of information and techniques, including machine learning techniques, BLAST, MEME (Multiple Em for Motif Elicitation)/MAST (Motif Alignment and Search Tool), and IgE epitope mapping to determine IgE-specific epitopes and allergen motifs [53]. BepiPRED 2.0 uses a Random Forest algorithm determined from crystallographic structures. Residues with thresholds higher than 0.5 were used to determine linear B cell epitopes [54]. The ultimate consensus epitope derived from a combination of software prediction and presented in all species were considered as the cross-reactive B cell epitopes.

### 4.3. Prediction of T Cell Epitope

The identification of T cell epitopes is principally based on the exploration of peptide fragments that bind to the MHC class II. NetMHCIIpan-4.0 (https://services.healthtech.dtu.dk/service.php?NetMHCIIpan-4.0; accession date: 19 February 2022) was used to predict HLA-DR-based T cell epitope in the regions of HLA-DR DRB101, HLA-DR DRB103, HLA-DRB301, HLA-DRB401, and HLA-DRB501. NetMHCII-2.3 was also applied to predict the HLA-DQ binding epitope. Those included HLA-DQA10101-DQB10501, HLA-DQA10102-DQB10602, HLA-DQA10301-DQB10302, HLA-DQA10401-DQB10402, HLA-DQA10501-DQB10201, and HLA-DQA10501-DQB10301. Both methods predicted peptide binding to any MHC class II of a known sequence using Artificial Neural Networks (ANNs), and the candidates were grouped as either a strong binder (≤2 percentile range), weak binder (percentile rank ≤ 10), or no binder (>10 percentile rank) [55]. As a result, the ultimate consensus sequences of predicted epitope obtained from the HLA-DR-based T cell epitope and HLA-DQbased T cell epitope prediction were categorized as the cross-reactive B cell epitopes.

### 4.4. Structure Retrieval and Homology Modeling

Since the crystal structure of tropomyosin of all species in this study has not yet been established, all structures were either retrieved from the AlphaFold database or predicted by the ROBETTA server (Table 1). The 3D structures of proteins in AlphaFold DB (https://alphafold.ebi.ac.uk; accession date: 15 February 2022) were predicted using a deep-learning-based artificial intelligence program that combines both physical and biological information about protein structure and multisequence alignments to predict protein conformation, which has been ranked as the number one protein prediction server in CASP14 [22]. ROBETTA (https://robetta.bakerlab.org; accession date: 15 February 2022) was also used to model tropomyosin from species that cannot be found in the AlphaFold database. This server uses both physics- and energy-based force field types and Monte Carlo, a filtering protocol to choose the suitable ab initio models [56].

### 4.5. Structural Refinement and Validation

The GalaxyRefine2 server (https://galaxy.seoklab.org/cgi-bin/submit.cgi?type=REFINE2; accession date: 17 February 2022) was utilized to refine the 3D model obtained from the AlphaFold database and Robetta prediction tool. The selection of this refinement tool was based on QMEANDisCo score testing after structure refinement using several methods, involving GalaxyRefine server, DeepRefine, and PREFMD (data not shown). Initial structures were subjected to quality assessment by using PROCHECK [24], and ERRAT [57] in SAVES v6.0 to validate the predicted models. ProSA (https://prosa.services.came.sbg.ac.at/prosa.php; accession date: 25 February 2022) was also applied to validate the tertiary structure. This server employs the data of the inputted structure to calculate the overall stereochemical quality of the protein structure [58]. Finally, the QMEAN program was used to assess the global and local (per residue) quality of the predicted structures [25].

### 4.6. Discontinuous Epitope Prediction

Most of the B cell epitopes were determined to be discontinuous. For this reason, the refined model base in the PDB coordinate was subjected to ElliPro, an online server (http://tools.iedb.org/ellipro/; accession date: 28 February 2022) for structure-based prediction of antibody epitopes with the threshold score > 0.5. This software is a most comprehensive protocol containing three algorithms based on three-dimensional structure data to predict discontinuous epitopes as protrusion index (P.I.) values. Ellipro was shown to demonstrate the best performance and provided an AUC value of 0.732 [59].

## Figures and Tables

**Table 1:** Tropomyosin from different sources used in this study.

Allergen	Biological Source	Common Name	Uniprot Acession No.	Structure Sources
Mac r 1	*Macrobrachium rosenbergii*	Giant freshwater prawn	D3XNR9	Robetta
Pen m 1	*Penaeus monodon*	Black tiger shrimp	A1KYZ2	AlphaFold
Pen a 1	*Penaeus aztecus*	Brown shrimp	Q3Y8M6	Robetta
Der f 1	*Dermatophagoides farinae*	American house dust mite	Q23939	AlphaFold
Bla g 7	*Blattella germanica*	German cockroach	Q9NG56	Robetta
Per a 7	*Periplaneta americana*	American cockroach	Q9UB83	AlphaFold

**Table 2:** Consensus linear B cell epitopes identified by AlgPRED and BepiPRED.

			AlgPRED (IgE Epitope)		BepiPRED-2.0 (Linear B-Cell Epitopes)					
	Residue	Peptide ID	Epitope Sequence	Species Consensus	AHDM	AC	BTS	BS	GC	GFP
Group 1 overlapped peptides	238–252	B1	RAEFAERSVQKLQKE	All	/	/	/	/	/	/
	241–255	B2	FAERSVQKLQKEVDR	All	/	/	/	/	/	/
	243–259	B3	ERSVQKLQKEVDRLEDE	All	/	/	/	/	/	/
	244–258	B4	RSVQKLQKEVDRLED	All	/	/	/	/	/	/
	251–259	B5	KEVDRLEDE	All	/	/	/	/	/	/
Group 2 overlapped peptides	187–197	B6	ESKIVELEEEL	All	/	/	/	/	/	/
	190–204	B7	IVELEEELRVVGNNL	All	/	/	/	/	/	/
	193–207	B8	LEEELRVVGNNLKSL	All	/	/	/	/	/	/
	196–210	B9	ELRVVGNNLKSLEVS	All	/	/	/	/	/	/
	199–206	B10	VVGNNLKS	All	/	/	/	/	/	/
	199–213	B11	VVGNNLKSLEVSEEK	All	/	/	/	/	/	/
Unique 1	85–91	B12	VAALNRR	All	/	/	/	/	/	/
Unique 2	137–141	B13	DEERM	All	/	/	/	/	/	/
Unique 3	144–151	B14	LENQLKEA	All	/	/	/	/	/	/
Unique 4	169–183	B15	LAMVEADLERAEERA	All	/	/	/	/	/	X

AHDM, American house dust mite; AC, American cockroach; BTS, Black tiger shrimp; BS, Brown shrimp; GC, German cockroach; GFP, Giant freshwater prawn. / = epitope found in target species; X = epitope was not found in target species.

**Table 3:** Predicted linear T cell epitopes.

Epitope Prediction	MHCII Alleles	Peptide ID	Residues	Consensus Core Epitope	Binding Type
HLA-DQ	DQA1*101–DQB1*501		-	-	
	DQA1*102–DQB1*602	T01	166–174	ARKLAMVEA	Weak binder
	DQA1*301–DQB1*302	T02	188–196	SKIVELEEE	Strong binder
		T03	187–195	ESKIVELEE	Strong binder
		T04	167–175	RKLAMVEAD	Weak binder
		T05	169–177	LAMVEADLE	Weak binder
		T06	165–173	VARKLAMVE	Weak binder
		T07	173–181	EADLERAEE	Weak binder
		T08	186–194	GESKIVELE	Weak binder
	DQA1*401–DQB1*402	T05	169–177	LAMVEADLE	Strong binder
		T07	186–194	EADLERAEE	Strong binder
		T02	188–196	SKIVELEEE	Weak binder
		T06	165–173	VARKLAMVE	Weak binder
		T03	187–195	ESKIVELEE	Weak binder
		T09	204–212	LKSLEVSEE	Weak binder
	DQA1*501–DQB1*201	T05	169–177	LAMVEADLE	Strong binder
		T02	188–196	SKIVELEEE	Strong binder
		T05	169–177	LAMVEADLE	Weak binder
		T10	189–197	KIVELEEEL	Weak binder
		T11	253–261	VDRLEDELV	Weak binder
	DQA1*501–DQB1*301		-	-	
HLA-DR	DRB1*0101		-	-	
	DRB1*0301	T12	144–152	LENQLKEAR	Weak binder
		T13	190–198	IVELEEELR	Weak binder
		T14	172–180	VEADLERAE	Weak binder
	DRB3*0101		-	-	
	DRB4*0101	T05	169–177	LAMVEADLE	Strong binder
		T15	171–179	MVEADLERA	Strong binder
		T16	res8–16	MQAMKLEKD	Strong binder
		T09	204–212	LKSLEVSEE	Weak binder
		T11	253–261	VDRLEDELV	Weak binder
	DRB5*0101	T17	197–205	LRVVGNNLK	Strong binder
		T13	190–198	IVELEEELR	Weak binder

**Table 4:** Evaluation parameters for the tertiary structure of modeled tropomyosin from different allergen sources.

	Ramachandran Plot (%)				ERRAT Overall Quality Factor	QMEAN Score	ProSA Z-Score
	Residues in Favorable Regions	Residues in Allowed Regions	Residues in Generally Allowed Regions	Residues in Disallowed Regions			
Black tiger shrimp	100	0	0	0	100	0.78	−2.91
Brown shrimp	98.9	0.7	0	0.4	100	0.68	−4.94
Giant Freshwater prawn	99.3	0.4	0.4	0	100	0.66	−4.97
American cockroach	100	0	0	0	100	0.82	−2.5
American house dust mite	100	0	0	0	100	0.81	−3.01
German cockroach	100	0	0	0	100	0.82	−2.63

**Table 5:** Consensus discontinuous B cell epitopes identified by Ellipro.

ID	Sequences	Residues	Linear B Cell Epitopes (IgE Epitope)	Linear T Cell Epitopes
DB1	M [D or E] AIKK, M	1–7, 8	No	Yes (8; strong binder)
DB2	QAMKLEKDNA[M or I]D[R or C]	9–21	No	Yes (9–16; strong binder)
DB3	[L or N or I]R, E	34–35, 37	No	No
DB4	[A or I or T], [DE or EE or VE], K, [S or G]	69, 72–73, 76, 83	No	No
DB5	V, EK	209, 212–213	Yes	Yes (209, 212; weak)
DB6	[T or I], [TR or AK or NK]	227, 230–231	No	No
DB7	AR, E, RS, KL, E, RL	237–238, 240, 244–245, 248–249, 252, 255–256	Yes (238, 240, 244–245, 248–249, 252, 255–256)	Yes (255–256; weak)
DB8	Vary	258–262, 264–265	Yes (258–259)	Yes (258–261; weak)
DB9	Vary	268–284	No	No

## Data Availability

The data presented in this study are available on request from the corresponding author.
